# Achievements and challenges in developing health leadership in South Africa: the experience of the Oliver Tambo Fellowship Programme 2008–2014

**DOI:** 10.1093/heapol/czx155

**Published:** 2018-07-08

**Authors:** Jane Doherty, Lucy Gilson, Maylene Shung-King

**Affiliations:** 1 School of Public Health Building, University of the Witwatersrand, (Education Campus), 27 St Andrew's Road, Parktown, Johannesburg, South Africa; 2 Division of Health Policy and Systems, School of Public Health and Family Medicine, Faculty of Health Sciences, University of Cape Town, Anzio Road, Observatory, Cape Town, South Africa; 3 Department of Global Health and Development, Faculty of Public Health and Policy, London School of Hygiene and Tropical Medicine, Keppel Street, London, UK

**Keywords:** Evaluation, capacity building, human resources, management

## Abstract

The Oliver Tambo Fellowship Programme is convened by the School of Public Health and Family Medicine, University of Cape Town, South Africa. It is a health leadership training programme with a post-graduate Diploma at its core, supplemented by management seminars, mentorship and alumni networking. An external evaluation was conducted in 2015 for the period since 2008. This rapid, descriptive study made use of mixed methods—including a document review of existing Programme material (management reports, anonymized alumni’s implementation project reports, exit interviews, field interviews and e-mailed questionnaires), a brief e-mailed questionnaire, and 18 semi-structured telephonic interviews conducted by the evaluator with Programme alumni, convenors and senior government line managers. Data were analysed according to indicators and associated criteria developed by the evaluator on the basis of the Programme’s objectives, international experience, the nature of the South African health system and the particular philosophy of the Programme. The evaluation found that the Diploma offered a unique contribution. This is because it sought less to convey new technical knowledge, than to empower and galvanize students to become change agents in the complex settings of their workplaces. Reflective practice was an important part of this process. Alumni were able to point to a number of positive changes in their management practice and motivation, translating these into improved performance by their teams and more effective health services. Alumni also helped to build the capacity of their own and other staff, sharing the knowledge and skills they had gained through the Programme, and leading by example. However, the Programme found it difficult to arrange adequate mentorship or peer support for alumni once they returned to their workplaces, pointing to the need for human resource development units in government to become more active in supporting alumni and holding them accountable for improving practice.

**Key Messages**

- Innovative health leadership training makes a vital contribution to leadership capacity development in low- and middle-income countries.
- Reflective practice is an essential skill for aspirant leaders as it stimulates personal growth.
- Modern leadership for the public health system requires team-building and joint decision-making, and this can be modelled through appropriate leadership training techniques.
- Government human resource development units need to partner with training institutions to support leadership development, including ongoing mentorship for newly trained leaders.

## Introduction

Recent literature identifies poor leadership as one of the most important problems impeding health systems development in low- and middle-income countries (Preker *et al.* 2006; World Health Organisation 2007; Daire *et al.* 2014; Galaviz *et al.* 2016; World Health Organisation and Alliance for Health Policy and Systems Research 2016). However, strategies to strengthen leadership are under-researched (Burgoyne *et al.* 2004; Curry *et al.* 2012; Doherty *et al.* 2013; Doherty 2014; Reich *et al.* 2016a). Leadership training programmes have also found it difficult to support newly trained leaders once they return to the complex and challenging workplaces characteristic of the health sector: this is especially so in poorly resourced settings, but is also found in high-income countries (Kebede *et al.* 2012; Doherty *et al.* 2013; Doherty and Gilson 2015; Erol *et al.* 2015; Nakanjako *et al.* 2015).

New generation training programmes are therefore experimenting with techniques both to prompt behaviour change more effectively and encourage long-term mentorship and other support for health leaders as they grapple with the myriad challenges of their working day (Management Sciences for Health 2008; Kebede *et al.* 2010, 2012; Edmonstone and Robson 2014; Nakanjako 2015; Galaviz *et al.* 2016; Reich *et al.* 2016a). Central to new approaches to leadership training is the importance of reflective practice, the modelling of appropriate behaviours and strengthening teamwork (Curry *et al.* 2012; Doherty 2013; Daire *et al.* 2014; Doherty and Gilson 2015; World Health Organisation and Alliance for Health Policy and Systems Research 2016). Workplace-based training is receiving increasing attention as an effective mechanism to supplement formal, residential training at academic institutions, and sometimes as an alternative (Doherty and Gilson 2015; Edmonstone 2015; Nakanjako *et al.* 2015).

This article contributes to the emerging literature on these new generation training programmes by presenting the findings of an evaluation of The Oliver Tambo Fellowship (OTF) Programme at the University of Cape Town in South Africa for the period 2008–2014. This Programme was initiated in 1996 and named after the President of the African National Congress who had died shortly before the first democratic elections in 1994. At that time, the Programme was geared towards the most senior public health managers responsible for transforming South Africa’s fragmented, apartheid-era health services into a unified, equitable health system (over the years, training has evolved to support district and facility managers charged with implementing policy as well).

While this article assesses the Programme’s achievements between 2008 and 2014, its main focus is to draw lessons for others seeking to offer leadership training programmes in low- and middle-income countries by considering the philosophy and techniques that lay behind its successes. In addition, the purpose of discussing the challenges faced by the Programme, and the on-going efforts to respond to them constructively, is to help others address similar problems in their own environment. Gaining greater clarity on effective mechanisms for developing senior managers and leaders is important for inculcating effective leadership practices and building a strong health system in low- and middle-income countries, especially in preparation for the implementation of universal health coverage reforms.

## The activities and philosophy of the OTF Programme

The OTF Programme evolved out of a Postgraduate Diploma in Health Management that is still convened by the School of Public Health and Family Medicine at the University of Cape Town. For the period 2008–2014, and following the first external evaluation in 2005 (Boufford and Crisp 2005), the Programme was offered jointly with the University’s Graduate School of Business, which took responsibility for coordinating two of the Diploma’s four modules. In line with developments internationally, this period saw a shift of focus from management to leadership training, underpinned by an understanding of health systems as complex systems, characterized by what Grint (2005) calls ‘wicked’^1^ problems and requiring new, innovative leadership skills (Gilson and Daire 2012; Uhl-Bien *et al.* 2007; Gilson 2016). This shift took time and the pedagogical approach and content of the Programme evolved as the convenor sought, first, to understand which interventions worked best and, second, to systematize these interventions.

For the period under review, students were nominated for the Programme by the South African Departments of Health (both national and provincial), as well as a city health department in one of the nine provinces, but they also had to meet the academic requirements of the university. The majority of students graduated within 18 months of enrolment.

The four residential modules (three of 8 days and one of 5 days) were run over a year. Students completed a range of assignments between each module, always entailing personal reflection, critical thinking skills and diagnosing and addressing challenges specific to their own workplaces. Together these modules focussed on understanding health systems as complex systems, the nature of complex systems, the politics of change within health systems and in policy implementation, and on developing systems thinking skills and practices. A final management project that was larger in scope and implemented over the 4 months following the last module, required considerable reflection, planning, implementation and adjustment over time, of a set of small-scale interventions designed to suit the specific context of their workplaces. A key element within and between each module was a focus on personal reflective practice—with dedicated time set aside for experiential work on this in each module and a series of personal reflections required between modules.

For students on the OTF Programme in this period, the Diploma was augmented by mentoring in the workplace, participation in a growing alumni network and a number of other alumni support and development activities (including the creation of an electronic platform for newsletters and notices, seminars and meetings and an alumni survey). These activities sought to encourage workplace-based learning and interaction, and reinforce the strong focus of modules on the application of new knowledge, skills and behaviours to participants’ specific working environments.

The objectives of the OTF Programme were (and still are): to strengthen the South African health system by supporting health managers (particularly those within the public sector) to develop the practices and behaviours of strategic and effective leaders who generate public value; and to develop a network of health managers (again, particularly within the public health sector) through which exchange of experience and knowledge can take place. The concept of achieving ‘public value’ (Moore 1995; Benington 2009) is integral to the Programme, and emphasizes not only the importance of health as an outcome of health systems, but also the role that health systems play in society at large. Health systems act as safety nets for the most vulnerable in society, and influence how people perceive themselves to be valued by society at large as well as overall trust in government (Gilson 2003). The notion of public value relates closely to the South African government’s commitment to equity and transforming the health system in the post-apartheid era (Republic of South Africa 2003), as well as the more recent commitment to universal health coverage—by improving financial protection and access to required health services of good quality—through a proposed National Health Insurance scheme (Department of Health 2015).

Box 1 presents the OTF Programme’s analysis of the leadership needs of the South African health system, developed over time and through engagement with the students in the Programme (see also Doherty and Gilson 2011; Gilson and Daire 2012). As this analysis reveals, the underlying theory of effective leadership that the Programme espoused during the evaluation period is that it builds on, and acts through, sound interpersonal relationships that, in turn, are informed by appropriate values and ethics: this is in alignment with relevant international thinking, especially around complex systems (Uhl-Bien *et al.* 2007; Daire *et al.* 2014; Chunharas and Davies 2016). Effective leaders use such relationships to build support for, and strengthen the legitimacy of, their programmes of action, and also manage the array of stakeholders affected by their efforts to achieve health system transformation (Gilson 2016). As an OTF Programme document from the evaluation period explained in more detail,

*‘The key element of effective leadership is the ability to build and sustain relationships. Good leaders demonstrate professional and ethical behaviour, motivation and sense of purpose in all their activities. They enthuse and motivate their staff, creating positive environments for team working in which team members support each other. They demonstrate the legitimacy of their activities, and develop constituencies that support them, by securing support from their own senior managers and drawing on wider networks of people and organisations in fulfilling their mandates. Finally they are also able to manage and defuse conflict. In these ways effective leaders build trust with others and by building this trust, support the achievement of their organisational goals’ (Oliver Tambo Fellowship Programme 2008).*

Box 1. The OTF Programme‘s analysis of leadership requirements for the South African health system, developed over time and through engagement with the students

- Leadership is distinct from, although related to, management. Poor leadership is a particular weakness of the South African health system and is one of the explanations for poor strategic and operational management, even where resources are adequate
- Leaders are not just the people who are officially designated as managers. In addition, they occur at all levels of the health system. It is important to build ‘distributed leadership,’ essentially connecting leaders across the system in productive ways
- Public health sector leadership needs to be informed by a set of values reflecting the social objectives of the country. These values need to be internalized by leaders so that they infuse their daily activities
- An important responsibility of leaders is to develop a vision for their institutions that harnesses—and prioritizes—efforts in support of this vision
- Another important aspect is the development of productive interpersonal relationships with staff and other stakeholders as it is through people that organisational change is achieved. Productive interpersonal relationships are founded on respect for others as well as an understanding of one‘s own strengths and weaknesses. Often they include managing bad relationships and interpersonal conflict in productive ways
- In the context of these leadership needs, hierarchical and rule-governed relationships are ineffective. To be effective leaders of change, managers have to involve staff and other stakeholders in decision-making processes, actively facilitate joint decision-making, and encourage staff and stakeholders to understand one another‘s viewpoints and experiences. Team-building and conflict management skills are therefore important skills of a good leader
- Effective communication is another key component of this style of collaborative leadership
- Health systems are extremely complex, especially as some are nested within one another. Each management problem is consequently located within inter-acting systems and a specific context. Leaders therefore need to be problem-solvers who employ analytic techniques that take account of the interactions between many components of the health system, understand the constraints and opportunities of contextual factors and appreciate that people—with their own understandings and interpretations—form part of this dynamic mix
- Problem solving is therefore not just about dealing with technical issues but also about thinking through how to influence and shape people‘s behaviours and responses. This includes careful framing of messages, for example, and careful thinking about how to manage different power relationships
- Leaders do need to have relevant, technical competence as well as these broader leadership skills
- Dynamic leadership requires continual reflection in order to build upon strengths, adjust strategies where they are ineffective and develop innovative solutions. Leaders therefore need to be lifelong learners who lead cycles of action-learning (essentially ‘learn through doing’)
- Leaders need to develop personal resilience in order to deal with the daily challenges of working in complex bureaucratic organisations

_____________________________________________________________________________________________________________________

Sources: Adapted by the evaluator from various reports of the Oliver Tambo Fellowship Programme and personal communications with course convenors

During the evaluation period, the OTF Programme developed a training strategy that sought to develop effective leaders of this sort. This strategy addressed both the ‘soft’ areas of personal reflection and self-knowledge, and inter-personal relationship development for health system transformation, as well as the ‘hard’ skills and tools to analyse problems, set priorities and take action in complex systems. In combination, these were intended to equip students with both the capacity and the confidence to address leadership challenges.

**Figure 1.**
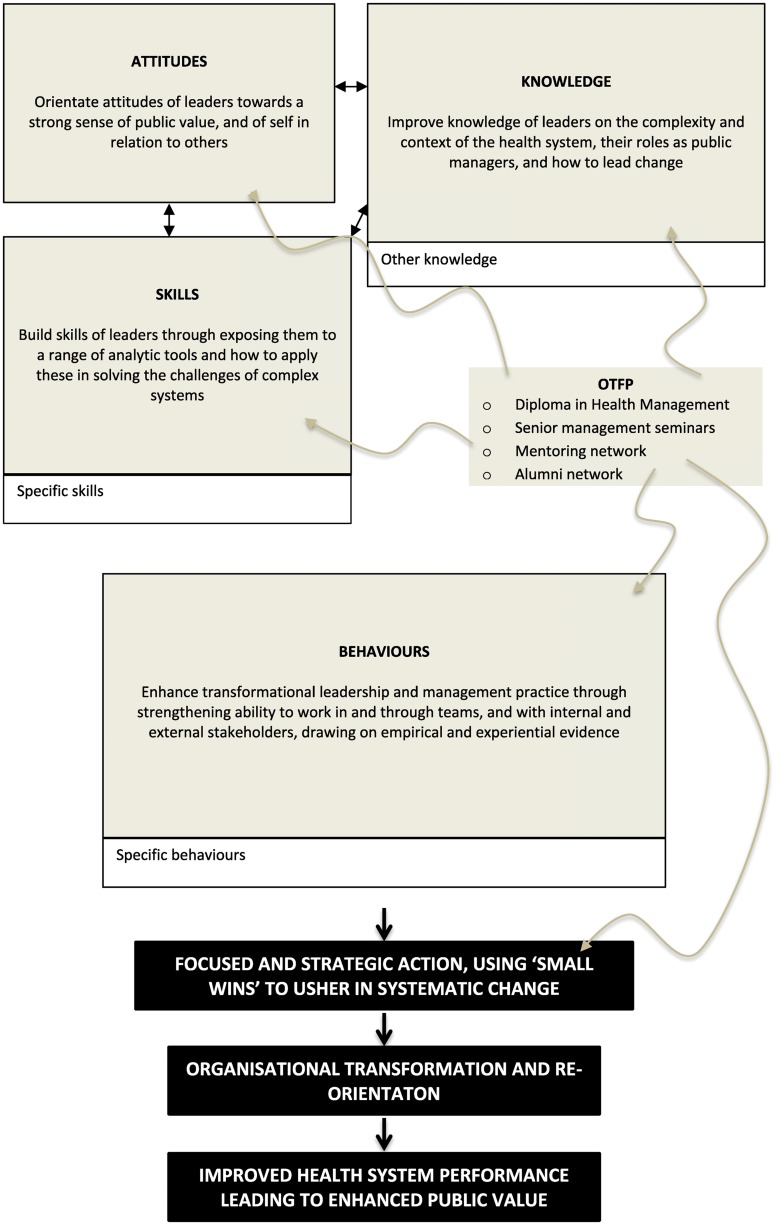
The strategy of the OTF Programme in supporting effective leadership. Note: Clear boxes represent knowledge, skills and behaviours that leaders also need but which were not prioritized by the OTF Programme as they did not represent the core attributes of good leaders and were relatively easy to acquire through other training opportunities. Sources: compiled by the evaluator from various reports of the Oliver Tambo Fellowship Programme, and personal communications with course convenors

Figure 1 explains the strategy by showing where and how the OTF Programme chose to intervene, using grey arrows and shading. It shows that the Programme intervened strongly in shaping the *attitudes* of students through allowing them to focus on themselves and become mindful of the consequences of their actions on the health system.

While it understood that effective leaders and managers need to acquire a wide range of *knowledge*, the Programme chose to focus on transmitting that aspect of knowledge that assists leaders to intervene strategically at any level of the health system. This is not about the usual understanding of strategic planning and management, but about the strategic use of interventions that are likely to leverage and spread change. This is important, as the aim of leadership training is not simply to amass knowledge, but to be able to make it actionable (Uhl-Bien *et al.* 2007; Gilson 2016). The assumption of the Programme was that more detailed and technical knowledge—such as an understanding of labour relations legislation or the details of financial management—could be acquired through other means (this is why the ‘knowledge’ box in Figure 1 is only partly shaded).

The OTF Programme spent a lot of effort in transmitting a particular set of generic *skills* to students that could be adapted and applied by leaders in any situation or setting. It assumed that, with these particular skills, students would be well equipped to deal with the ever-changing dynamics of complex health systems (Uhl-Bien *et al.* 2007).

Equipped with relevant attitudes, knowledge and skills, the Programme encouraged students to develop a ‘personal framework of practice’ that allowed them to act in, and on, the health system through a range of positive *behaviours*. It encouraged students to adopt and practise these behaviours, and to adjust them through reflection and on-going learning. It emphasized the need to achieve ‘small wins’ (Weick 1984) as first steps in the achievement of systematic change, and to focus on increasing public value as the raison d’être of health systems transformation (Management Sciences for Health 2008). An important feature of the Programme was its emphasis on leaders working with, and through, others—whether ‘above’ or ‘below’ in the formal hierarchy—through the creation of dynamic teams. In some senses then, the Programme sought to combine elements of workplace based practice learning within a formal course-based approach to training (Edmonstone 2015).

## Methods

The external evaluation on which this article is based was conducted in 2015 and examined the period since the first evaluation (i.e. 2008–2014). It was conducted by an independent researcher who is the first author of this article (JD). The convenors of the Programme played no role in the evaluation itself, but are the co-authors of this article (LG, MSK). This is in line with the focus of the article, which is to convey the philosophy and techniques of the course, as well as some of the changes adopted by the Programme since the evaluation, as opposed to simply the evaluation results. Indeed, the evaluation was always intended to be formative, contributing to the further development of the Programme, rather than to simply assess whether it had achieved its past objectives.

The evaluation was a rapid, descriptive study that made use of mixed methods techniques. First, a document review was conducted of existing material that had been generated by the convenors of the Programme. This included: 18 Programme reports and other management documents; a random sample (31 documents) of material reflecting alumni’s post-graduation views of the Programme (through exit and subsequent interviews that had been conducted by the convenors over the years); 72 management project implementation reports that had been written by alumni as part of their training; and a quantitative analysis of 41 existing field interviews and e-mailed questionnaires with respect to alumni’s assessment of the extent to which the Programme had met its objectives.

Second, a brief questionnaire was e-mailed by the evaluator to all 91 alumni who had enrolled in or after 2008, and graduated by 2014 (this was the period examined by the evaluation). The purpose of the survey was simply to ascertain whether alumni were still working in South Africa (and in which sector and province). The survey had an 84% response rate.

Third, the evaluator conducted 18 semi-structured telephonic interviews, mainly with Programme alumni (half of whom were selected purposively and half of whom were selected randomly). The two Programme convenors and three senior government line managers who supervized Programme alumni were also interviewed.

In order to assess outputs, outcomes and impacts, the evaluator developed indicators of success based on an understanding of new developments in leadership training internationally, the requirements of the South African health system (as laid out in Box 1) and the particular philosophy and objectives of the Programme itself, as communicated through Programme documents and conversations with the convenor (and summarized in Figure 1). Tables 1–3 present the indicators and criteria that were developed.^2^ They reflect Kirkpatrick’s well-known approach to evaluating training programmes which recognizes ‘four levels’ of influence—how the programme makes participants feel, what participants learn on the programme, how the ‘on-the-job effectiveness’ of participants is affected and, finally, how the effectiveness of the business (or service) in which the participants operate is changed (Kirkpatrick and Kirkpatrick 1994).

**Table 1:** Assessment of the outputs of the OTF Programme

Outputs (the production of graduates from the Programme, senior management seminars, and the activities of the mentoring and alumni networks)		
Criteria	Indicators	Assessment
**Effectiveness**		
The Programme meets its overall objectives	The Programme regularly produces adequate numbers of graduates	++
	The Programme has regular mentorship activities	+
	There is a regular programme of senior management seminars	+
	The alumni network has regular activities	+
	Graduates are satisfied with the Programme overall	+++
The overall structure and content of the Programme activities are appropriate	The Programme is well structured in that it a) transmits an appropriate range of knowledge and skills and b) provides an appropriate balance between theoretical and practical experience	+++
	Students are exposed to a range of learning opportunities	+++
	There is an appropriate range of networking and mentorship activities	+
	Assessments are appropriate and marked fairly	+++
Teaching staff/supervisors/mentors have the appropriate knowledge and skills	Trainers have the appropriate knowledge and skills relating to the content of courses and seminars, and are skilled in appropriate teaching methods	+++
	Mentors have the appropriate knowledge and skills	+
	The convenor has good coordination skills	+++
Students feel able to get support when necessary	Lecturers make sufficient time available to their students and students feel comfortable approaching them for help	+++
	Students are able to get advice from lecturers while away from the University of Cape Town	++
	Mentors provide support to students in terms of their personal growth and problems, guide them through the training process and act as role models	+
Students have good access to physical and other resources that support learning	Students have good access to computers, internet, relevant books and journals, and working spaces, and the University as a whole provides a supportive and enabling environment	+++
The Programme is continually monitored and periodically evaluated	The Programme is monitored regularly by the Programme coordinators	+++
	A Board oversees the Programme	+
	The Programme is evaluated by external experts	+++
**Relevance**		
The Programme meets the needs of students	There is a high demand for the Programme amongst students and their institutions	++
	Students actively seek out the Programme because of its key characteristics and ability to further their chosen careers	++
The Programme meets the needs of employers	Employers recommend that their employees apply for the Programme	+++
The Programme is aligned to country and regional capacity-building priorities	Students acquire knowledge and skills that are identified as scarce and important for health management	+++
	Government and donors acknowledge the Programme as relevant	+++
**Efficiency**		
The Programme uses inputs wisely	The selection process targets students who are well-suited to the course	++
	Students graduate within a reasonable time period	+++
	Daily management of the course is efficient	+++
	Alumni network coordination is efficient	(++)
	There is an efficient use of funds for mentorship and networking activities	(++)
**Sustainability**		
The Programme is likely to be able to continue	Students and their families are able to afford the costs of the Programme	+++
	The convenors are able to retain suitably qualified staff to co-ordinate the course, lecture, supervise and mentor	+++
	The convenors are able to recover the staff and other costs associated with teaching and supervision	+++
	There are funds and staff to continue the coordination of the OTF Programme	(+++)

Note: more ‘+’ signs indicate greater success in meeting a criterion (with a range from one to three ‘+’ signs), whereas brackets around a ‘+’ indicate that this indicator is difficult to assess given the complexity of the issue.

**Table 2:** Assessment of the outcomes of the OTF programme

Outcomes (staff retention, how alumni felt as a result of the training they had received, and changes to on-the-job effectiveness of alumni)		
Criteria	Indicators	Assessment
Alumni are recruited into and retained in the South African public health sector	Alumni find employment (or are promoted) as a result of the knowledge, skills, attitudes and behaviours developed through the Programme	+++
	Alumni are retained in South Africa	+++
	Alumni are retained in their original provinces	+++
	Alumni are retained in the public health sector	+++
Alumni are in jobs that have potential to impact on the health system	Alumni are in positions where they have influence over the functioning of the health system	+++
Alumni are able to impact positively on the health system	Alumni feel empowered by the Programme to implement management transformation because they have: greater understanding of the nature, requirements and responsibilities of a manager’s job generally, and of a public health manager’s job as a policy implementer awareness of their particular roles within the broader health system in supporting health system performance and delivering public value enhanced reflection on their management and leadership style and awareness of their limitations as a leader internalized a problem-solving and learning approach to their work, including the use of a range of technical tools better understanding of human behaviour and individual differences, and the importance of staff behaviour and attitudes in the performance of a facility improved persuasiveness in arguing for new interventions improved self-confidence and assertiveness in carrying out their managerial responsibilities recognition of their own limits and able to ask for advice and support resilient and able to persevere despite encountering obstacles	+++
	Graduates feel motivated to implement management transformation	+++
Graduates employ more effective leadership styles	Graduates demonstrate the following characteristics of transformational leadership: greater focus on developing a guiding strategy and coordinating and motivating staff, rather than simple administration improved communication with colleagues and other staff increased and more effective involvement of team members in collaborative decision-making greater attention to developing sound interpersonal relationships with other policy actors and managing stakeholders strategically greater ease with initiating uncomfortable conversations innovative and practical responses to solving problems, including addressing implementation challenges on the ground consideration of the full range of factors contributing to a situation effectively delegate responsibilities and authorities to their subordinates they are able to give fair, objective and useful feedback on the performance of their staff	+++
Alumni have an enhanced sense of personal pride and job satisfaction	Alumni feel proud of the changes they have made to their leadership styles	+++
	Alumni have received recognition from colleagues and line managers for their improved leadership	+++
	Graduates enjoy their jobs	( ++)

Note: more ‘+’ signs indicate greater success in meeting a criterion (with a range from one to three ‘+’ signs), whereas brackets around a ‘+’ indicate that this indicator is difficult to assess given the complexity of the issue.

**Table 3:** Assessment of the impacts of the OTF programme

Impacts (how health services change as a result of the actions of alumni, and changes to the overall management capacity of the public health sector)		
Criteria	Indicators	Assessment
Alumni impact positively on the performance of the health institutions in which they work	Health organisation’s management practices change through the transformational leadership provided by alumni	++
	Health services improve as a result of interventions by alumni	++
Health management and leadership capacity development is institutionalized in South Africa	Alumni build health management and leadership capacity within their own institutions (including training and mentoring young managers)	+++
	Alumni work collaboratively to build health management and leadership capacity across the public sector (including training or mentoring young managers, as well as fostering networking)	++

Note: more ‘+’ signs indicate greater success in meeting a criterion (with a range from one to three ‘+’ signs), whereas brackets around a ‘+’ indicate that this indicator is difficult to assess given the complexity of the issue.

Documents, interview transcripts and notes were analysed according to themes that had been previously identified on the basis of the objectives and approach of the Programme, as well as the indicators. Data from different sources were triangulated to enhance the validity of the information, with enormous consistency noted in the information generated from different sources.

Ethical approval for the study was granted by the authors’ institution and the free and informed consent of all subjects was obtained.

There are several limitations to the evaluation. First, the size of the study precluded more formal measurement of the outcomes and impacts of the Programme (such as workplace-based measurements).

Second, to deal with some of the problems of bias associated with asking alumni to reflect on their own performance, the study was originally designed to include interviews with four senior government officials unrelated to the course. It was also intended to interview 20 alumni (15 randomly sampled and 5 purposively) to ensure a full spectrum of experience. However, it proved difficult to access such senior and busy people. As a result, only 14 alumni were interviewed (while an additional one sent very brief comments). Of these, only seven could be randomly sampled. Despite these limitations, the conclusions of all the key informants, and the extensive document review, were remarkably similar and the evaluator is of the opinion that the study was reaching saturation, even with only 15 interviews. In fact, it was impossible to analyse all the student and alumnus feedback documents submitted by the Programme coordinators (estimated to be around 200 in number) and it is likely that there is more corroborating evidence than presented in the evaluation.

Third, while the response rate for the alumni survey was very high, which reflects the strong connection that alumni still have with the Programme, the questions were designed to find out how well alumni had been retained in the public health sector and in their original province: it may be precisely those who could not be contacted who were not retained. Therefore, the analysis of the survey presents a range within which the retention rate is likely to fall, depending on whether or not missing alumni are categorized as having left the public health sector.

Finally, it is inherently difficult to measure the impacts of a training programme on a complex health system that is subject to a myriad of other influences and constraints. This is because it is difficult to identify and measure the chain of events that lead to service change, and attribute elements of service change to changed leadership and management practices on the part of individuals, let alone assess whether these changed practices were as a result of participation in the training programme. The findings relating to impacts are therefore necessarily tentative.

## Results

Tables 1–3 attempt to summarize the considerable detail amassed by the evaluation, and rate the Programme’s achievements according to the indicators and criteria identified by the evaluator (the more plus signs against a criterion, the more success the Programme had in this area, whereas brackets around a plus sign indicates that this area was difficult to assess, given the complexity of the issue). A summary of this nature is not easily able to convey the nuances of a Programme that, according to the extensive feedback provided by alumni, is very successful. The presentation of the results in this section is therefore confined to the main points and interested readers need to examine the Table in order to understand achievements in more detail.

Further, in order to be rigorous, Tables 1 and 2 judge many of the dimensions assessed quite strictly. To balance this, it is important to emphasize the enthusiasm for the Programme expressed by its alumni. To give but two examples, one alumnus said that ‘The basic strength was unleashing the potential to realize that your characteristics, your experiences, are actually worthwhile … It opened you up to yourself, to the world around you and how much is possible’ while another said that ‘you know, that culture of learning, I could feel it, I could experience it, I could practically feel it and live it. You know, the intensity of the Programme there, it’s quite, quite awesome.’ This general enthusiasm for the Programme must be borne in mind when considering the challenges raised below.

### The outputs of the OTF Programme

Table 1 examines the outputs of the Programme, namely, ‘the achievement of targets for the production of services’ (Molund and Schill 2007) or, in the case of this study, the production of graduates, senior management seminars and the activities of the mentoring and alumni networks. As discussed earlier, the indicators and associated criteria used were developed in response to the Programme’s objectives, international experience, the nature of the South African health system and the training philosophy of the Programme. With respect to outputs, these indicators and criteria were grouped according to whether they assessed the effectiveness, efficiency, relevance or sustainability of the Programme (Molund and Schill 2007).

The OTF Programme produced over 250 graduates between its inception and 2014. However, the annual output of 15 new graduates during the period 2008–2015 was relatively small, and there was a worrying dropout rate amongst those who originally enrolled, of 25%. While personal problems played a part in this pattern, it also appears to have been due to the difficulty and demanding workload of the Programme, both during the residential modules and the inter-modular periods.

Some of the managers reported that they had had significant changes to their scope of work midway during the course and these changes (due partly to significant restructuring in the public sector, redeployment and the assumption of multiple portfolios), coupled with the demanding nature of the course, proved overwhelming for them. This was especially so where students’ line managers and workplaces were reportedly not very accommodating of inter-modular study needs. In addition, course convenors reported that some students were not appropriately selected and were mismatched to the course. This was partly as a result of the somewhat arbitrary nomination of students to the course by some government departments.

As already noted, generally, alumni expressed considerable satisfaction with the structure, content and teaching style of the course. Many alumni referred to certain key concepts and strategic thinking tools as revolutionizing their thinking and problem-solving abilities.

The variety of tools that they found particularly helpful included policy analysis, systems thinking and tools, critical thinking, stakeholder analysis and engagement, reflective practice, and the concept of achieving ‘small wins’ when initiating change. Alumni particularly valued tools that enhanced their understanding and application of systems thinking (derived from the business environment) and a health systems approach (derived from the public health environment). These tools provided them with a starting point for unpacking the challenges they faced in their specific contexts, a mechanism for drawing their management team into discussions about potential solutions, and the confidence to tackle what had initially seemed overwhelmingly complex problems.

Alumni also expressed appreciation for the adult learning principles underlying the course and the related teaching techniques. The practice of extensively using small-group work was identified as particularly effective by alumni, as they felt that it exposed them to the experience of people from different disciplinary backgrounds and different levels of the health system. Alumni also highlighted the practice of frequently having to present three-minute verbal summaries of work as another technique that was very useful in facilitating learning and also simulated the realities of the working environment where, in meetings, as managers they only have a brief chance to put forward their position and ideas.

From the perspective of the course convenors, other essential and effective training approaches included: the focus on teaching as facilitation; helping students to share their wisdom and learn from one another; role-modelling the breaking down of traditional teaching (and health system) hierarchies in classroom interactions; facilitation styles that embraced adult learning; and recognizing course participants as peers. The use of real world case studies and problem-based learning in the classroom allowed for immediate application of new knowledge and skills. Additional approaches include the generation of questions for further reflection; the use of experiential learning approaches to encourage self-reflection; and invitations to health system managers from outside the course to share their experiences. Critically, the practice-linked assignments required the application of new knowledge and skills to actual workplace challenges and contributed to team and workplace strengthening, as reflected on by a number of participants.

The other outputs of the OTF Programme—the alumni network (including senior management seminars) and the mentorship programme—were less successful in achieving their intended objectives than the Diploma, despite concerted efforts by course convenors. A key common reason was that both the Programme, and the health services from which students came, were not well-staffed. This meant heavy workloads for academic staff, workplace-based mentors and students alike, and made it difficult for people to put time aside to meet and interact. Course convenors, in particular, were not able to be as active in these areas as they would have liked.

With respect to the mentorship component, the expectations of mentors and students did not necessarily coincide, especially for those mentors who did not have in-depth knowledge of the content and pedagogical approach of the Programme. Training to remedy this problem was provided to workplace-based mentors by the Programme in the earlier years. Nonetheless, the Programme conveners concluded at one point that ‘mentoring, as a voluntary activity, is a rather hit and miss activity—with potential for support where, for example, there is mutual effort, personal links, a particular focus of discussion, proximity to allow more regular engagement’ (School of Public Health and Family Medicine 2011). On reflection, dedicated staff were probably required to support the development of a more effective mentorship system.

Alumni reported that difficulties in implementing formal mentorship support were aggravated by the fact that the government departments from which students came, generally did not seem to monitor students’ progress on the course, nor track them once they had graduated. Alumni were therefore not held accountable for implementing what they had learned. Equally importantly, they were not given active support in practising the skills they had acquired, nor some leeway to make mistakes while experimenting with new management approaches. The implications of this for future development of the Programme are discussed later.

An alternative strategy of peer mentoring was adopted in later years. The majority of students valued the support they received from fellow students. They typically extended their peer-network beyond the formally allocated peer-mentor pairs. Some of these relational networks endured beyond graduation, as indicated by an alumnus: ‘we still have a social media group going almost 5 years now’. However, these developments stopped short of setting up ‘action learning sets’, whereby alumni meet together regularly as workplace-based teams (with or without other leaders and managers) as a mechanism for learning and solving complex problems jointly, because of a variety of constraints, as described later.

### The outcomes of the OTF Programme

Table 2 examines the outcomes of the Programme, namely, ‘the short and medium term effects on the attitudes, skills, knowledge or behaviour of groups or individuals’ (Molund and Schill 2007) or, in the case of this study, the changes in the leadership and management behaviour and practice of alumni. These include retention of alumni in the public sector, how alumni felt as a result of the training they had received, and self- and peer-reported changes to on-the-job effectiveness of alumni.

Of the alumni who were tracked by the evaluation, all had remained in South Africa and 91% had been retained in the public health sector and in the same province (if all of the 22 alumni who could not be tracked had left the public sector, this percentage would be closer to 70%, but the figure is unlikely to be this low). The view of one senior manager who reflected on this retention success was that the Programme,

*‘has contributed towards the developing of high level and good quality managers for the health sector and the main thing is that they have stayed … in the public sector, with very few exceptions … I think people who were contemplating leaving started believing that things could change, that they could stay and make a difference and change even those things that were frustrating with the environment … and that’s why people stayed because quite a lot of people arrived at the OTF with a sense of despair and they were looking at other options.’*

Given that the OTF Programme graduates remained in the public sector, generally in senior positions, they had the potential to impact positively on the health system by employing more effective leadership styles. The evaluation investigated whether this was indeed the case in detail. The various sources of information revealed that the time spent on the Programme had a transformative effect. Alumni confirmed that the Programme had indeed provided them with an array of analytic tools that they could use to understand the environments in which they worked, identify the root causes of problems faced by their management teams, strategize around how to shift their organisations towards improved effectiveness, engage more constructively with staff and stakeholders, and embark on the first steps towards context-specific change.

As illustrated by the array of quotes in Box 2, the application of these tools was couched within an understanding of the policy development and implementation process, and a commitment to serving communities and meeting social objectives, so that alumni could re-align their efforts with broader health system goals.

Box 2 Some instances where graduates claimed their interventions had led to changed leadership and management practices in their institutions

‘We have established a core team for the chief directorate that includes even senior hospital managers to discuss policy matters that will impact on the hospitals mostly. It ha[s] increased the team cohesion as the hospitals now feel that they are part of the provincial office team. We have also introduced the weekly reports to assists in monitoring hospital[s] on a more regular basis.’

‘I have established a forum where the Directorate meets with labour organisations so that feedback and inputs from nurses and staff may be shared to allow me consider different perspectives in my planning. I have managed to break the walls of silo mentality as I incorporate the soft systems methodology in my system. I now include other directorates in my plans and activities … The benefits are tremendous.’

‘The team that worked with me in planning the interventions and implementing the answers had initially experienced the process [as] very lengthy … As we went on, I was able to coach them on techniques and analysis methods to make the interventions credible and ensuring proper planning. We all have learned that a problem situation does have much more perspectives than we have thought and the many different angles for approaching the concern, learned from this program, provided a clear insight into the different identified variables and the necessary interventions required.’

‘This process has improved the team work especially with the senior managers in finance. The process has highlighted the issues that need to be resolved in our financial management, and the process has succeeded in streamlining our thought processes and also giving us direction on how to deal with the problems.’

‘The understanding of policy implementation has improved service delivery in the district. In the past we were just implementing programmes through experience and really did not read the policies in detail. Now all the managers co-ordinating primary health care programmes have been motivated by me to read their policies and also monitor if they are in line with what is supposed to be implemented. This has gain [sic] results as the district was among the district[s] with the best management of Tuberculosis and oral health services. This recognition was given to us by National Health.’

_____________________________________________________________________________________________________________________

Source: Interviews conducted by the evaluator with Programme alumni

Very importantly, alumni noted that the acquisition of these skills occurred in tandem with a process of deep personal reflection which led alumni to understand their roles, strengths and weaknesses as leader-managers, transform their interpersonal relationships, continually learn from their failures and successes, and bring a sense of energy and resilience to their management practice. Indeed, a changed perspective on interpersonal relationships, informed by a better understanding of their own personalities and behaviours, was one of the most consistent outcomes mentioned by alumni who were interviewed in the study. Many said that the OTF Programme had led them through a profound self-examination, as people and as leaders. This had led them to question the way they engaged with other people, whether it was staff that reported to them, supervisors to whom they reported, or stakeholders with whom they had to work to achieve common objectives. They learnt patience, an appreciation of different perspectives and the importance of involving others in decision-making. Above all, they learned to listen and communicate more effectively. Consequently, alumni felt that they had become better people as well as better people-managers. The series of quotes in Box 2 reveal how this led to the development of teams that were more effective at solving problems encountered in the workplace.

### The impact of the OTF Programme

Table 3 looks at whether the OTFP led to ‘longer term effects or the effects at the scale of societies, communities or systems’ (Boufford and Crisp 2005)—in other words, whether the positive outputs and outcomes of the Programme had a positive impact on the South African health system itself. The Table looks at whether health services changed as a result of the actions of alumni, and whether there were changes to the overall management capacity of the public health sector.

Reference was made earlier to the difficulty of establishing this conclusively in a complex environment. In analysing the responses of OTF Programme alumni, it became clear that they work in a variety of settings in different provinces and at different levels of the health system. They answer to managers with different characteristics and manage different sorts of teams. They themselves differ in terms of personality, attitude and ability. They all face a variety of political, financial and administrative obstacles to implementing change.

Further, alumni expanded on the many additional issues that make the health system environment in which they operate complex, unwieldy to manage and generally unreceptive to innovation (see Box 3). These included political interference that distorts priorities, poor senior leadership capacity due to inappropriate appointments, the lack of a culture of excellence and critical mass of good managers, the problem of senior managers who feel threatened by strong people under them and exert a predominantly ‘command and control’ style of leadership, and resistance from other public sector managers and staff as a result of bureaucratic inertia or lack of exposure to the concepts and skills that alumni had acquired on the Programme. These points resonate with other analyses of contextual factors impeding change in South Africa (Gilson and Daire 2012; Doherty 2014; Doherty and Gilson 2015), and reflect the international literature characterizing health system leadership challenges as complex and ‘wicked’ (Grint 2005).

Box 3 Environmental and institutional barriers to implementing change identified by OTF Programme alumni

- Budget constraints and shortage of human resources and supplies
- Political interference that distorts priorities
- Poor senior leadership capacity due to inappropriate appointments, including cadre deployment
- Failure of senior policy-makers to include all stakeholders leading to contestation
- An unwieldy and inflexible bureaucracy, including centralized authority
- The ‘silo mentality’ where staff in different sections work independently and do not collaborate
- A high turnover of staff
- Red tape that slows down appointments
- High administrative loads
- Lack of a culture of excellence and critical mass of good managers
- Lack of authority to implement change (e.g. if working at the national level)
- Senior managers who want to ‘lead from the front,’ feel threatened by strong people under them and exert a predominantly ‘command and control’ style of leadership
- Resistance from other public sector managers and staff as a result of bureaucratic inertia, the lack of a culture of excellence or lack of exposure to the concepts taught on the OTF Programme

_____________________________________________________________________________________________________________________

Source: Interviews with Programme alumni

It would be expected, therefore, that alumni would have varying levels of success in improving service delivery and inculcating transformational leadership and management practices into the public health system. Nonetheless, many graduates, and the few senior officials interviewed, were able to point to some positive changes in terms of health system performance (see Table 4), although time constraints prevented the reviewer from verifying these claims. Alumni were also able to provide evidence of their success in building the management and leadership skills of their management teams through sharing the approaches they had learned on the OTF Programme. For example, one alumnus described her leadership approach to overcoming bureaucratic inertia thus:

**Table 4:** Improvements to health services as a result of actions by graduates

Type of change	Evidence of improved services
**National-level interventions**	
Improving the impact of support visits to facilities and provinces for administration of the National Tertiary Services Grant	Some sites began to submit their reports in time for deadlines.
**Provincial-level interventions**	
Implementation of joint planning at a provincial Department of Health	The planning process was shifted from silo to joint, integrated planning, including the formation of an Inter-cluster Forum
Improving the implementation of a bursary scheme	Bursaries were awarded and monitored at the district rather than provincial level, and involved communities in decision-making
Addressing problems with nursing services in a province	A skills audit was conducted which identified a lack of supervision skills. A supervision tool and guidelines for nurse managers was developed
Reducing diarrhoea rates	The implementation of door-to-door campaigns in collaboration with the water affairs department led to a drastic reduction of diarrhoea rates in the province, following mentorship of staff in programme implementation
Readying public and private hospitals for collaboration in a province	A public-private hospital CEO forum was established, together with the development of a Commitment Charter. A collaborative project with an NGO was initiated. Effort was put into changing the private sector‘s perception that public sector management is sub-standard. These interventions were effective in impacting positively on public-private trust levels and subsequently the level of collaboration increased significantly from the original situation of sporadic, unstructured public-private interactions
Improving the availability of drugs at a pharmaceutical depot	Within three weeks, the availability of fast-moving items was up from 73 to 84%
Improving the turnaround time of lab results by provincial National Health Laboratory Services	A communication plan to improve communication between clinical and laboratory managers was implemented at a pilot hospital. Results indicated that adherence to turnaround times increased from 70 to 85% in 2 months
Improving health and safety in forensic pathology services in a province	Health and Safety officers were appointed at all 18 mortuaries, and Health and Safety committees were set up in all four regions. The number of occupational injuries in 1 month declined from 3.2 to 2, and the number of work days lost through sick leave declined from 39 to 12 per month
**District-level interventions**	
Improving a district health information system	Data capturers were appointed in all sub-district facilities. A data quality assessment team was appointed to do monitoring and evaluation
Improving the quality of a district health information system through training nurses in data capture	At facility level, registered nurses were trained on the completion of daily data sheets. At the sub-district level, one of the data capturers was delegated to be responsible for the sub-district information. At senior management level, the chief director instructed that this approach should be presented in all the districts
Improving the information system in a sub-district	After the intervention, 80% of the clinics submitted the information to the health information officer by the due date. 50% of clinic managers used the health information to make decisions. This was a big improvement over the previous situation. All participants agreed that effective communication improved in the sub-district and that staff were being informed of what was expected of them. The number of complaints per week was used to monitor the improvements: the complaints reduced
Streamlining the communication from a sub-district office	The e-mail and fax communication system was reviewed and standardized. This has rendered lost documents almost a thing of the past
An assessment of patient information documents and records management in preparation for a health expenditure review	Data requirements and registers were streamlined. An evaluation of two clinics showed some improvements in terms of alignment of data reported at the province and the source documents
Improving the support given by a sub-district management team to facility managers	A sub-district management team was established, a sub-district planning session was hosted and the management team supported a facility manager towards the implementation of a new and innovative idea
Improving support services to sub-district and specialized services	A staff recruitment and retention strategy was implemented that led to major improvements. The evaluation of the process revealed that staff were satisfied with the availability of labour broker staff when a high workload was experienced. They also indicated that they were very happy with the implementation of scarce skills allowances
Improving supply chain processes in a district	The visible impact included reduced procurement errors, reduced waiting times for orders and deliveries, reduced complaints from cost centre managers and end-users, improved transparency, reduced irregular expenditure and less time spent on processing orders. More time was spent on strategic management issues resulting in improved effectiveness
Improving interaction with city departments that were involved in health and sanitation in informal settlements	Relationships have improved which makes monitoring more effective. It has become possible to explain, using the concept of systems, why certain interventions have had very different consequences from intended. Some of the recommendations were incorporated into tenders and maintenance strategies
Improving the patient transport system in a district	The improvement in the efficiency of the transport system could clearly be seen by the decrease of wasted seats over time
Ensuring that HIV-positive patients who are eligible for TB treatment in a province actually receive it	Record-keeping was improved and the format of the register for tracking patients was revised. Developmental partners and others observed an uptake in treatment during an evaluation of the intervention
**Interventions in hospitals**	
Improving waiting times in a district hospital	A number of interventions were carried out: identification of a queue manager for channelling clients to the right queues; a client relations officer for resolving problems regarding the service; and a courtesy officer for assisting the aged, disabled and any other clients that needed help. To ensure the outpatient’ area was clean by the time the service started in the morning, and to avoid unnecessary delay, after-hours allocations were made for general assistants to clean the outpatients’ department and nurses to prepare the consulting room. The way the pharmacy was designed was changed: pharmacy assistants were made responsible for replenishing drugs so that pharmacists could concentrate on issuing prescriptions and getting the queue to move faster. Continuous monitoring of waiting times was initiated
Improving waiting times at an hospital outpatients’ department	In a short period of time a significant difference was made. The average waiting time at reception reduced
Improving waiting times at an hospital outpatients’ department	A good patient flow was created from entry to consulting the health care practitioners. Time spent by staff in the corridor writing names in the register was eliminated. The number of clients consulted in the morning increased
Reduction of pharmacy waiting times at a hospital	A significant improvement in the reduction of the waiting times was reported when the management introduced the system of queue marshals as well as an express queue
Improving supply-chain processes in a district hospital	An acting Supply Chain Manager was appointed whilst an interim Bid Committee was established. It seemed that some headway was made in decreasing over-expenditure
Getting facilities accredited	Three facilities were accredited
Improving staff satisfaction at a hospital radiology department	Sorting out the format of meetings, promoting the vision of the department, informing staff about the organogramme and the lines of authority, and training and delegating to radiographers, contributed to an improved service and greater job satisfaction. Waiting times decreased (for some procedures from 55 to 30 days). A follow-up survey showed that more staff were satisfied with their jobs (an increase of 38–66%)
**Interventions in clinics**	
Better resourcing of clinics	PHC clinics are better staffed and equipped because of the application of the allocative efficiency principle in moving budgets from less to more beneficial areas
Extending clinic hours	A clinic has begun to offer after-hour services (which are funded) as the after-hour services at the hospital are not accessible to many people because of distance
Reducing ART defaulters at a community health centre	There was a general increase of around 8% to 10% of ART defaulters placed back on treatment
Improving the TB cure rate	The TB cure rate went up from around 80–90%. In one of the clinics that had as much as a quarter of the cases, the cure rate had only been 60% but went up to 85%

Notes: This Table only quotes project reports or interviews that explicitly described direct impacts on actual service delivery. There were many other projects that probably impacted on service delivery but did not describe this clearly enough, or would have had indirect impacts (for example by undergoing in-depth analyses of the problems confronting their institution and identifying needed actions). Impacts were achieved over a matter of weeks rather than months, given the requirements of the OTF Programme.

*‘You push when people are ready, you push when people feel the need … I test the things in my turf where I have control and I offer them when I think people are now interested … and sometimes we’re under pressure and there’s a problem that needs solving and they’re looking for an answer.’*

## Discussion

The Programme’s approach, and the lessons it has learned in developing this approach over the evaluation period, provide some guidance to others seeking to develop leadership transformation programmes elsewhere, especially in low- and middle-income settings. Particular innovations in the South African context were a focus on: leadership rather than management; practice rather than knowledge (encouraging implementation of management changes, supported by cycles of reflection and adjustment); space for peer engagement, support and relationship-building in the classroom (when there is so little space for this in the health system at large); and continual internal evaluation of the Programme by students and alumni. These innovations are increasingly recommended for new leadership training approaches and documented elsewhere (Uhl-Bien *et al.* 2007; Curry *et al.* 2012; Doherty 2013; Daire *et al.* 2014; Doherty and Gilson 2015; Reich *et al.* 2016b; World Health Organisation and Alliance for Health Policy and Systems Research 2016).

However, the Programme is still grappling with the challenge of providing post-training support to alumni back in the workplace. This is a common problem of university-based training programmes seeking to impact positively on health system performance (Doherty *et al.* 2013; Doherty and Gilson 2015; Edmonstone 2015; Erol *et al.* 2015). The Programme convenors did not find it easy to engage effectively with government human resource development departments or health management teams to find ways to harness the potential of new graduates. This is because the human resource function within the South African government relates more to personnel administration than to strategic human resource development and management. Consequently, human resource units rarely think strategically about whom they nominate for leadership training, or how to pro-actively support individuals’ career development and add value to the health system through training (e.g. by sending teams for training). Likewise, performance management systems do not protect time for students to study, or monitor the impact of their training on their own performance. Within this context, it is particularly difficult to envisage how to set up innovative, workplace-based training support, such as the action learning sets mentioned earlier. Indeed, the complex workplace dynamics—particularly, power imbalances—revealed through students’ experiences indicate environments that may limit the learning and action resulting from action-learning processes (Vince 2008). Nonetheless, the Programme is investigating lessons from other settings, including how to manage the challenges of facilitating action-learning to support productive engagements (Doherty and Gilson 2015; Erol *et al.* 2015; Nakanjako *et al.* 2015; Rigg and Trehan 2004).

Another major challenge facing South Africa (and other low- and middle-income countries) is how to inject greater numbers of highly trained managers into the health system more rapidly. It is difficult under current resource constraints to get to the point where a ‘critical mass’ of well-trained leaders can shift the organisational culture of the public sector, although fortunately there are more leadership training courses available in South Africa than two decades ago. The recent shift towards online or blended courses may result in less contact time away from work, but at the same time loses the strong relational and networking components that the OTF Programme alumni found particularly valuable. A critical challenge is how to encourage workplace-based learning on a regular basis as a complement to formal training (Doherty *et al.* 2013; Doherty and Gilson 2015; Edmonstone 2015).

In this light, finding ways to reduce the drop-out rate of students entering leadership programmes is a priority, particularly where the reason for dropping out is an overly heavy workload in the workplace. The OTF Programme has repeatedly tried to make the work requirements of the course more explicit for prospective enrolees and their supervisors, and seek commitment from their line managers for time to be set aside for study between modules, yet these efforts have been only partially successful. The rigour of the OTF Programme is one of its greatest strengths, and the opinion of the evaluator (JD) is that the academic standard of the Programme should not be compromized by shortening contact time. It does require ongoing assessment of the Programme content and workload, however, and ensuring a good alignment with the demands of an ever-changing health system.

It is probably beyond the scope of a relatively small course to put in place extensive mentorship and networking activities on a national scale. There is evidence that the use of practice-based methods within a structured programme places bigger demands on facilitators and requires a greater investment of time (Edmonstone and Robson 2014). In addition, the problem of isolation that newly trained managers often experience reflects problems in the health system that are bigger than one training programme can shoulder on its own. These include the general under-training of the management cadre, the weakness of human resource development efforts within the public sector, limited monitoring of the impact of training on its employees by government and the lack of a wider ‘community of practice’ for health managers. This points to the need for greater leadership by government itself with respect to a vision and practical strategy for leadership development in the health system. In this regard, it is a positive move that the South African government initiated the development of a South African Academy for Health Care Management and Leadership in 2012. One of the issues that such an Academy could explore is creating a balance between formal, qualification-based approaches and workplace-based leadership training initiatives (Edmonstone 2015).

Perhaps the OTF Programme should re-frame its role in supporting mentorship and alumni networking as building skills in government departments around supporting and monitoring new graduates to ensure that they use what they have learned in the workplace. This could possibly be done with one or two interested provinces in order to create ‘good practices’ that could be shared with other provinces, including experimenting with ways for alumni to support one another better, supporting line managers to enable alumni’s on-going development, and encourage the development of experienced mentors. This would require innovative thinking around how to support workplace-based learning as part of the routine practice of line managers.

## Conclusion

The conclusion of this evaluation was that the Oliver Tambo Fellowship Programme, as implemented between 2008 and 2014, was perceived by alumni to be a high-quality and effective leadership training programme, with a unique approach. Its alumni characterized participation in the Programme as a transformative experience and displayed a commitment to applying what they learned in the field. They also demonstrated a clear sense of belonging to a ‘community of practice’, with a particular ethos and shared approach to dealing with the complex challenges in the workplace.

Nonetheless, the Programme faces continuing obstacles that are common to many country settings. Indeed, training for health leadership is a complex task that poses many challenges to trainers. While the need to provide training that reflects the real—and unpredictable—world of health systems is well-acknowledged, mechanisms for doing so require further exploration, especially in low- and-middle-income countries where opportunities for workplace-based support tend to be limited. Further research on appropriate training mechanisms, and continual evaluation of leadership training programmes, are required to ensure that they evolve to meet the practical needs of leaders on the ground.

## Footnotes

1 Writing about the workplace challenges with which leaders deal, Grint (2005) characterizes them as ‘wicked’ rather than ‘tame’, in that they are complex, interact dynamically with the environment, exist across components of the health system and government, are rooted in historical and social phenomena, and are often intractable. This is what requires skills distinct from those of managers, who tend to deal with implementing known solutions to problems that, although often complicated, are more easy to contain.

2 The following definitions were adapted from Molund and Schill (2007): outputs are *‘the achievement of targets for the production of services’* and, in this study, represent the production of graduates from the Programme and the activities of the alumni network; outcomes are ‘*short and medium term effects on the attitudes, skills, knowledge or behaviour of groups or individuals’* and, in this study, are the changes in the leadership and management behaviour and practice of alumni; impacts are *‘the longer term effects or the effects at the scale of societies, communities or systems’* or *‘the totality of the effects of a development intervention, positive and negative, intended and unintended, from the point of view of target groups and other primary stakeholders,’* and, in this study, are the consequences for health systems, especially the public health sector.
